# HERC2 regulates RPA2 by mediating ATR-induced Ser33 phosphorylation and ubiquitin-dependent degradation

**DOI:** 10.1038/s41598-019-50812-x

**Published:** 2019-10-03

**Authors:** Yongqiang Lai, Mingzhang Zhu, Wenwen Wu, Nana Rokutanda, Yukiko Togashi, Weixin Liang, Tomohiko Ohta

**Affiliations:** 1 Department of Translational Oncology, St. Marianna University Graduate School of Medicine, Kawasaki, Japan; 2 Department of General Surgery, The People’s Hospital of Gaoming District of Foshan City, Foshan city, Guangdong province China; 3 Present Address: Oncology TA Division/Research & Development, AstraZeneca Japan, Osaka, Japan

**Keywords:** DNA damage response, Stalled forks

## Abstract

Replication protein A (RPA) binds to and stabilizes single-stranded DNA and is essential for the genome stability. We reported that an E3 ubiquitin ligase, HERC2, suppresses G-quadruplex (G4) DNA by regulating RPA-helicase complexes. However, the precise mechanism of HERC2 on RPA is as yet largely unknown. Here, we show essential roles for HERC2 on RPA2 status: induction of phosphorylation and degradation of the modified form. HERC2 interacted with RPA through the C-terminal HECT domain. Ubiquitination of RPA2 was inhibited by HERC2 depletion and rescued by reintroduction of the C-terminal fragment of HERC2. ATR-mediated phosphorylation of RPA2 at Ser33 induced by low-level replication stress was inhibited by depletion of HERC2. Contrary, cells lacking HERC2 catalytic residues constitutively expressed an increased level of Ser33-phosphorylated RPA2. HERC2-mediated ubiquitination of RPA2 was abolished by an ATR inhibitor, supporting a hypothesis that the ubiquitinated RPA2 is a phosphorylated subset. Functionally, HERC2 E3 activity has an epistatic relationship with RPA in the suppression of G4 when judged with siRNA knockdown experiments. Together, these results suggest that HERC2 fine-tunes ATR-phosphorylated RPA2 levels through induction and degradation, a mechanism that could be critical for the suppression of secondary DNA structures during cell proliferation.

## Introduction

One of the most noteworthy features of cancer cells is their genomic instability, which can largely be attributed to an aberrant response to replication stress; this is increased in cancer cells with activated oncogenes and accelerated cell growth. Genomic instability is further linked with cancer aggressiveness. Various proteins direct DNA replication to protect against such unfavorable consequences. Of particular note is replication protein A (RPA), which is an essential protein complex that is involved in mammalian DNA metabolic processes including DNA repair, recombination, and telomere maintenance in addition to replication^1^. RPA binds to and prevents the spontaneous annealing and formation of the secondary structure of single-stranded DNA (ssDNA). During replication stress, uncoupling of DNA polymerases and replicative helicases at stalled replication forks generates ssDNA regions, which are promptly bound by RPA^2,3^. ssDNA-bound RPA then acts as a platform to recruit and activate numerous proteins, including ataxia–telangiectasia and Rad3-related kinase (ATR), a member of the phosphoinositide-3-kinase-related kinases (PIKKs) which phosphorylates downstream targets to activate checkpoints, stabilizes stalled forks, and supports the completion of replication^3–5^.

RPA is a heterotrimeric complex of RPA1 (RPA70), RPA2 (RPA32), and RPA3 (RPA14)^1^. While RPA1 is a major subunit involved in DNA binding, both RPA2 and RPA3 are required for stability of the complex, and RPA2 is a major target for upstream kinases, including CDKs and the three PIKKs (ATR, ataxia–telangiectasia mutated [ATM], and DNA-PK)^6–8^. RPA2 comprises multiple critical Ser/Thr residues that are phosphorylated in sequential order in response to genotoxic stress. In a basic model, Ser23 and Ser29 phosphorylation by CDKs stimulated Ser33 phosphorylation by ATR, which in turn promoted Thr21, Ser4, and Ser8 phosphorylation by DNA-PK and, to some degree, ATM^9,10^. Although the order of phosphorylation varies according to the replication stress agent and cell cycle phase^10^, Ser33 phosphorylation is specifically mediated by ATR, and phosphorylation of Ser4/8 is the final event that produces the most hyperphosphorylated form of RPA2^9–12^. The hyperphosphorylation of RPA2 inhibits replication by preventing the association of RPA with the replication machinery and contributes to the recruitment of DNA repair proteins^13,14^. Meanwhile, ATR-mediated phosphorylation of RPA stimulates DNA synthesis and prevents ssDNA accumulation to alleviate low-level replication stress^15^.

While the phosphorylation of RPA2 has been extensively studied, how its protein stability is regulated remains enigmatic. In the present study, we show that the phosphorylation and protein stability of RPA2 are regulated by HERC2, a large HECT-type E3 ubiquitin ligase that plays critical roles in DNA replication and the damage response^16–19^. Recently, we showed that HERC2 suppressed G-quadruplex (G4) DNA by promoting the assembly and disassembly of RPA in the helicase complexes of BLM and WRN^20^. Inactivation of HERC2 E3 activity in cells inhibited the ubiquitination of RPA2 and caused RPA accumulation in the complexes, resulting in G4 accumulation and hypersensitivity to G4 stabilizers in cancer cells^20^. Here, we build on those results by further defining the essential roles of HERC2 in the degradation and ATR-mediated Ser33 phosphorylation of RPA2 in unstressed cells and cells under low-level replication stress. Functionally, HERC2 E3 activity has an epistatic relationship with RPA in G4 suppression when judged with siRNA knockdown experiments. Our results show that HERC2 regulates ATR-phosphorylated RPA2 levels through induction and degradation, a mechanism that could be critical for the suppression of secondary DNA structures during cell proliferation.

## Results

### HERC2 interacts with RPA through the C-terminal HECT domain of HERC2

**Figure 1:**
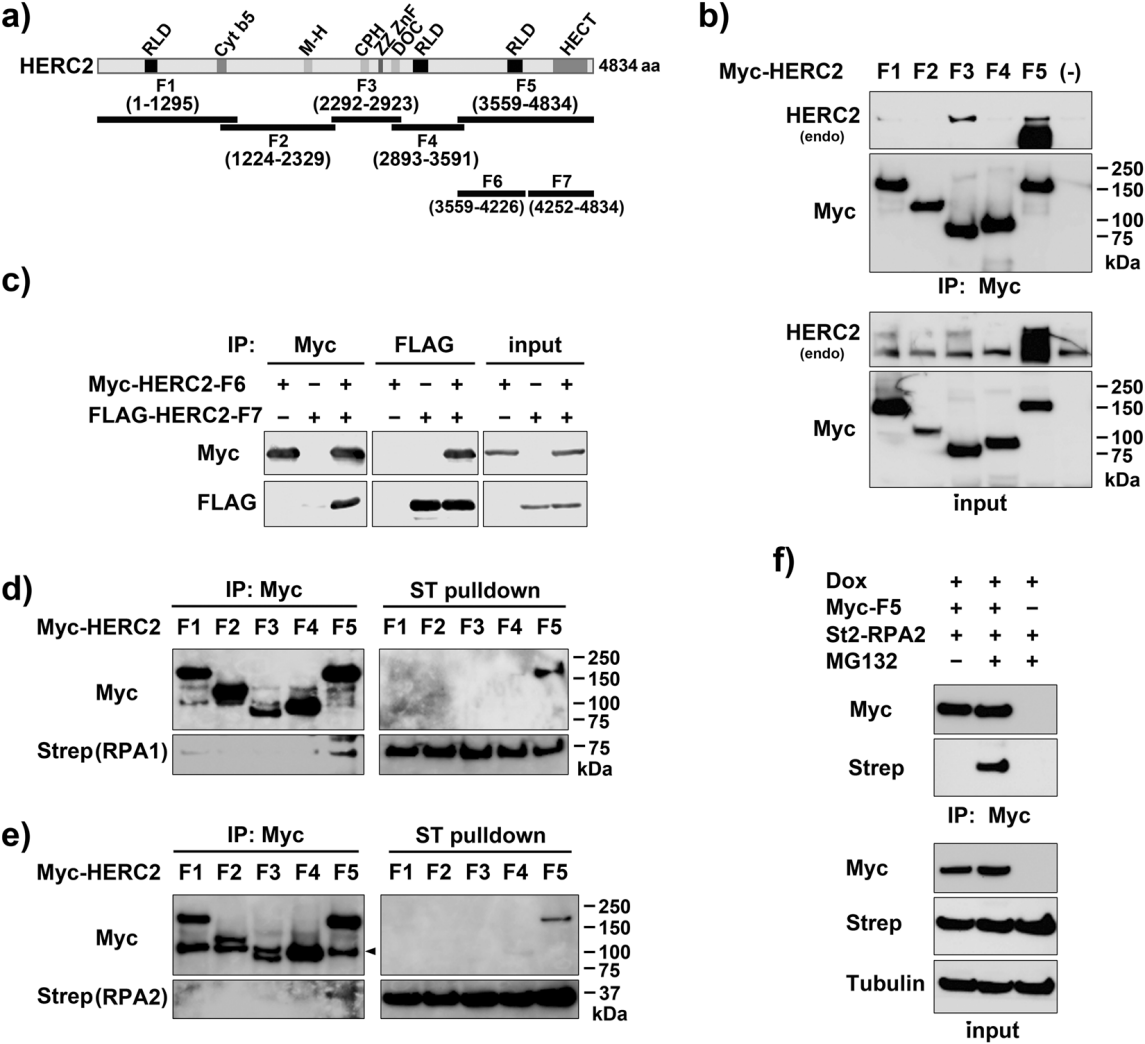
RPA interacts with the C-terminus of HERC2. (**a**) The indicated HERC2 fragments with an N-terminal Myc- or FLAG-tag were used to examine RPA binding and intramolecular binding of HERC2. (**b**) HEK-293T cells were transfected with Myc-HERC2 fragments. Interaction between the fragments and endogenous HERC2 was assessed by immunoprecipitation (IP) with anti-Myc antibody followed by immunoblotting (IB) with the indicated antibodies. Inputs were also loaded. (**c**) HEK-293T cells were transfected with the indicated plasmids and the interaction between fragments F6 and F7 was assessed by immunoprecipitation and immunoblotting with anti-Myc and anti-FLAG antibodies as indicated. (**d**,**e**) HeLa-shHERC2 cells were co-transfected with the indicated HERC2 fragments and St2-RPA1 (**d**) or St2-RPA2 (**e**) together with other RPA subunits (pEGFP-RPA1 or HA-RPA2, and FLAG-RPA3), induced with Dox, treated with MG132, and subjected to immunoprecipitation with anti-Myc antibody or Strep-Tactin pulldown followed by immunoblotting with the indicated antibodies. The arrow head indicates non-specific band from IgG. (**f**) HeLa-shHERC2 cells were co-transfected with Myc-HERC2-F5 and St2-RPA2 as indicated, induced with Dox, treated or not with MG132, and subjected to immunoprecipitation followed by immunoblotting with the indicated antibodies.

To investigate the functional link between HERC2 and RPA, we first attempted to map the site of RPA interaction on HERC2. Because HERC2 is an extremely large protein with 4834 amino acids, we employed immunoprecipitation followed by immunoblotting with exogenously expressed HERC2 fragments (Fig. 1a) in cells rather than using purified recombinant proteins. During the experiments, we noticed that two of the HERC2 fragments (F3; 2292–2923 and F5; 3559–4834) interacted with endogenous HERC2 (Fig. 1b). We further split fragment F5 and confirmed that residues 3559–4226 (F6) are capable of interacting with residues 4252–4834 (F7) by reciprocal immunoprecipitation and immunoblotting (Fig. 1c). Large HECT ubiquitin ligases often auto-ubiquitinate and oligomerize with their ubiquitin-binding domain in the C-terminal HECT domain, remaining poised for reactivation^21,22^. Therefore, the interaction observed could be due to this mechanism. Supporting this notion, residues 4252–4834 (F7) contained the conserved motif for the autoubiquitination site^21^. In addition, expression of the fragment F5 caused a smear band detected by anti-HERC2 antibody immunoblotting (Fig. 1b). The identity of this smear band is unknown at present. To avoid interaction with endogenous HERC2 and simplify the experiment, we depleted endogenous HERC2 when the HERC2 fragments were co-expressed with RPA in cells. HeLa cells stably expressing Dox-inducible HERC2 specific shRNA (HeLa-shHERC2 cells) were co-transfected with Myc-HERC2 fragments and St2-RPA1 or St2-RPA2 together with or without other RPA subunits and induced with Dox, followed by immunoprecipitation coupled with immunoblotting. However, we failed to produce any concrete results regarding the HERC2–RPA interaction (Fig. S1; data not shown). Because RPA2 is a possible HERC2 substrate for proteasomal degradation, we tested whether inhibition of proteasomes would increase our ability to detect the HERC2–RPA interaction. Indeed, immunoprecipitation with anti-Myc antibody and Strep-Tactin pulldown of either St2-RPA1 (Fig. 1d) or St2-RPA2 (Fig. 1e), co-expressed with other RPA subunits, reciprocally detected the HERC2–RPA interaction when cells were treated with the proteasome inhibitor MG132. St2-RPA2 was also co-immunoprecipitated with the HERC2-F5 fragments in an MG132-dependent manner without the co-expression of other subunits (Fig. 1f). Together, the results suggest that HERC2 interacts with RPA via its C-terminal HECT domain and that this interaction was enhanced by inhibition of proteasomes. Note that although detection of the interaction of the HERC2 fragment with RPA required MG132, interaction between endogenous HERC2 and RPA is constitutive and can be detected without MG132^20^.

### The C-terminal HECT domain of HERC2 ubiquitinates RPA2

**Figure 2:**
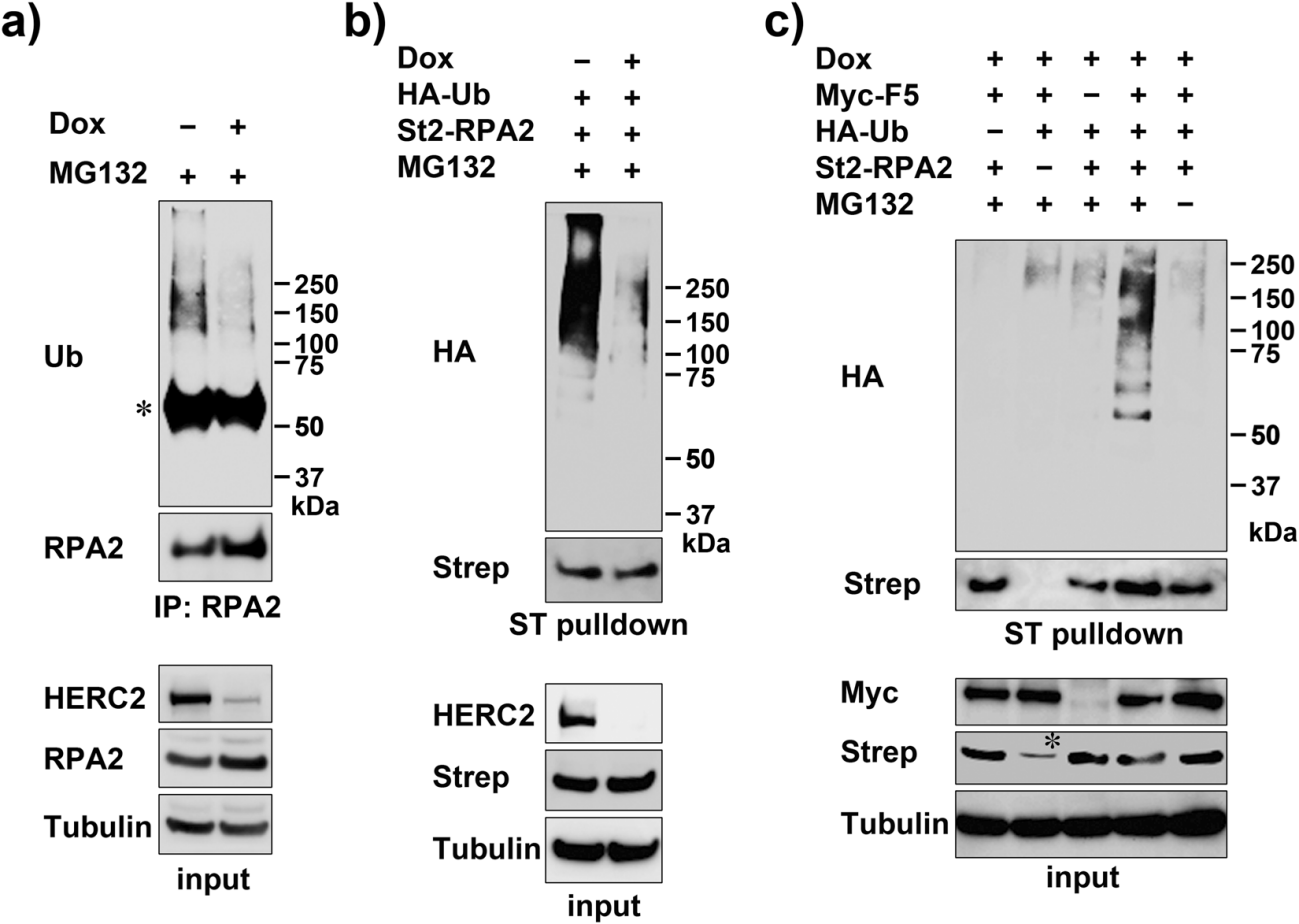
*In vivo* RPA2 ubiquitination mediated by C-terminus of HERC2. (**a**) HeLa-shHERC2 cells were induced or not with Dox, treated with MG132, and subjected to immunoprecipitation in denature condition followed by immunoblotting with the indicated antibodies. Inputs were also loaded. (**b**) HeLa-shHERC2 cells were co-transfected with St2-RPA2 and HA-ubiquitin (HA-Ub), induced or not with Dox, treated with MG132, and subjected to Strep-Tactin pulldown followed by immunoblotting with anti-HA antibody to detect ubiquitinated RPA2 products. (**c**) HeLa-shHERC2 cells were co-transfected with the indicated plasmids, induced with Dox, treated or not with MG132, and ubiquitinated RPA2 products were detected as in (**b**). The asterisk indicates non-specific band. Myc-F5: Myc-HERC2-F5.

Previously, we showed that endogenous RPA2 ubiquitination was abolished in HCT116 cells with E3-inactive HERC2^20^. To further confirm the ubiquitination of RPA2 by HERC2, we performed denaturing immunoprecipitation-based experiments to detect ubiquitinated RPA2 in different conditions. We tested the effect of HERC2 knockdown in HeLa cells and used different RPA2 antibodies for immunoprecipitation. Concordant with the previous result, endogenous RPA2 ubiquitination could be detected in a HERC2-dependent manner (Fig. 2a). Next, HeLa-shHERC2 cells were co-expressed with St2-RPA2 and HA-ubiquitin and treated with MG132. Ubiquitinated RPA2 products were then precipitated with Strep-Tactin pulldown and detected by immunoblotting with anti-HA antibody. Dox-induced depletion of HERC2 again dramatically reduced the ubiquitinated RPA2 products (Fig. 2b). Importantly, reintroduction of the HERC2-F5 fragment in the HERC2-depleted cells restored the ubiquitination of RPA2, which was detected in an MG132-dependent manner (Fig. 2c). The results confirmed that the C-terminus of HERC2 ubiquitinates RPA2 for proteasomal degradation.

### HERC2 is required for ATR-mediated phosphorylation of RPA2 at Ser33 induced by low-level replication stress

**Figure 3:**
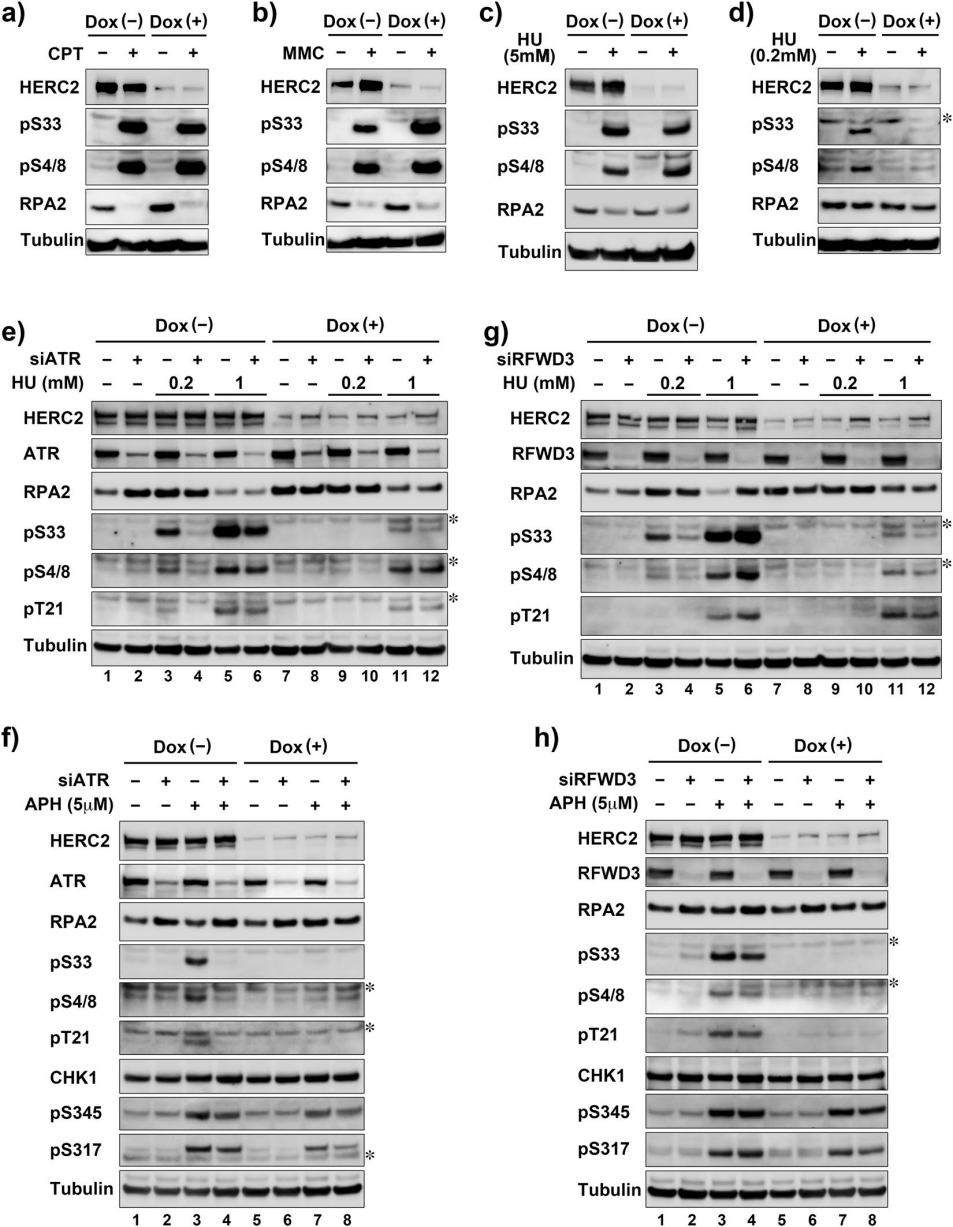
Depletion of HERC2 inhibit ATR-mediated phosphorylation of RPA2 induced by low-level replication stress. (**a**–**d**) HeLa-shHERC2 cells were induced or not with Dox, treated or not with the indicated genotoxic agents, and subjected to immunoblotting with the indicated antibodies. (**e**–**h**) HeLa-shHERC2 cells were transfected with siRNA specific to ATR (**e**,**f**) or RFWD3 (**g**,**h**) induced or not with Dox, treated or not with indicated concentration of HU (**e**,**g**) or APH (**f**,**h**), and subjected to immunoblotting with the indicated antibodies. The asterisks indicates non-specific bands.

RPA2 is phosphorylated at multiple sites by the PIKK kinases ATM, ATR, and DNA-PK in response to replication stress or DNA damage as described. We previously failed to detect the effect of HERC2 depletion on RPA2 phosphorylation at either Ser33 or Ser4/8^20^. Consistent with this, HERC2 depletion did not dramatically affect the phosphorylation induced by exposure to 5 µM CPT, 0.5 µg/ml MMC, or 5 mM HU for 16 h (Fig. 3a–c). However, we found that RPA2 Ser33 and Ser4/8 phosphorylation induced by 0.2 mM HU, the dose that remains permissive for DNA replication^23^, was inhibited by depletion of HERC2 either by Dox-induced shRNA (Fig. 3d) or siRNA transfection (Fig. S2a). Similar results were also observed in HERC2-depleted HeLa cells with a different shRNA targeting independent sequence in HERC2, arguing against off-target effects (Fig. S2b–d). Time course analyses suggested that HERC2 depletion continuously suppressed RPA2 Ser33 phosphorylation during 16 h exposure to 0.2 mM HU (Fig. S2e). RPA2 Ser33 phosphorylation induced by 6 h exposure to 5 µM APH, another replication stress that does not cause high level DNA damage, was also inhibited by HERC2 depletion (Fig. S2f).

To analyze whether the observed HERC2-dependent RPA2 phosphorylation is mediated by ATR, we tested the effect of combinatorial depletion of ATR and HERC2 on HU-induced RPA2 phosphorylation. HeLa-shHERC2 cells were transfected with control or ATR-specific siRNA, left untreated or induced with Dox, treated with 0.2 or 1 mM HU for 16 h, and subjected to western blotting (Fig. 3e). RPA2 Ser33 phosphorylation induced by 0.2 mM HU (lane 3), which was abolished by HERC2 depletion (lane 9), was inhibited by ATR depletion (lane 4), indicating that HERC2 is required for ATR-mediated Ser33 phosphorylation of RPA2. RPA2 Ser33 phosphorylation induced by 1 mM HU (lane5), which was dramatically reduced by depletion of HERC2 (lane 11), was also moderately inhibited by ATR depletion (lane 6). In contrast, neither RPA2 Ser4/8 nor Thr21 phosphorylation induced by 1 mM HU were affected by ATR depletion (lane 5 and 6), indicating that the phosphorylation was mediated independently of ATR by ATM or DNA-PK, likely due to DNA breakage as a consequence of stalled replication forks. In contrast, RPA2 Ser4/8 phosphorylation induced by 0.2 mM HU was inhibited by ATR depletion (lane 4), suggesting that this phosphorylation is induced after ATR-induced Ser33 phosphorylation as previously reported^9–12^. Interestingly, HERC2 depletion did not affect Ser4/8 and Thr21 phosphorylation induced by 1 mM HU (lane 11 and 12), indicating that HERC2 is required specifically for ATR-mediated phosphorylation of RPA2 in low-level replication stress and does not affect ATM- or DNA-PK-mediated phosphorylation of RPA2 after higher-order DNA breakage. Consistent results were also observed with a different siRNA targeting independent sequence in ATR (Fig. S3a).

The inhibition of replication stress-induced ATR-dependent RPA2 Ser33 phosphorylation by HERC2 depletion was also confirmed by 5 μM APH treatment (Fig. 3f). Consistent with the results of low-dose HU treatment, ATR-dependent Ser4/8 and Thr21 phosphorylation was also inhibited by HERC2 depletion under the condition. Similar results were also observed with the different ATR-specific siRNA (Fig. S3b).

Besides HERC2, two E3 ligases for RPA2, RFWD3, and PRP19, have been reported previously^24,25^. In addition to their roles in RPA2 ubiquitination, both E3s are required for RPA2 phosphorylation, in a similar fashion to HERC2. Therefore, we compared the impact of HERC2 with RFWD3 on RPA2 phosphorylation by combinatorial depletion. HeLa-shHERC2 cells were transfected with control or RFWD3-specific siRNA, left untreated or induced with Dox, treated with 0.2 or 1 mM HU for 16 h and subjected to western blotting (Fig. 3g). Depletion of RFWD3 dramatically reduced the RPA2 Ser33 phosphorylation induced by 0.2 mM HU (lane 4). However, after treatment with 1 mM HU, depletion of RFWD3 did not inhibit but rather enhanced the RPA2 Ser33 phosphorylation accompanied by enhanced Ser4/8 and Thr21 phosphorylation (lane 6). When compared to depletion of RFWD3, depletion of HERC2 more severely inhibited Ser33 phosphorylation (lane 9) and additional depletion of HERC2 canceled the increase in RPA phosphorylation by RFWD3 depletion (lane 12). The steady state level of RPA2, which was reduced after 1 mM HU treatment (lane 5), was recovered by RFWD3 depletion (lane 6), suggesting that DNA-PK/ATM-mediated hyperphosphorylated RPA2 was degraded by RFWD3. The reduced level of RPA2 after 1 mM HU treatment was also partially recovered by HERC2 depletion (Fig. 3e, lane 5 and 6 vs lane 11 and 12, and Fig. 3g, lane 5 vs lane 11), suggesting that HERC2 has a role in the degradation of RPA2 under the conditions. Although the detected reduction of RPA2 level could be caused by mobility shift due to phosphorylation, the recovery of the RPA2 level despite its hyperphosphorylation in RFWD3 depleted cells (Fig. 3g, lane 6) renders the possibility less likely.

We also compared the effect of HERC2 depletion on the phosphorylation of RPA2 induced by 5 μM APH with that of RFWD3 depletion. Under the test conditions, the effect of RFWD3 on RPA2 phosphorylation (Fig. 3h, lane 4) was much smaller than that of HERC2 (lane 7), indicating that HERC2 plays primary role on Ser33 phosphorylation rather than RFWD3 does. ATR also phosphorylates CHK1, one of the most vital substrates besides RPA2. We therefore explored whether depletion of HERC2 would affect CHK1 phosphorylation at Ser317 and Ser345, two major sites of ATR-mediated phosphorylation. Remarkably, depletion of HERC2 had almost no effect on CHK1 phosphorylation (Fig. 3f,h, lane 7), suggesting a substrate specific role of HERC2 in ATR-mediated phosphorylation. Interestingly, depletion of ATR only slightly inhibited CHK1 phosphorylation, while it completely inhibited RPA2 phosphorylation (Fig. 3f, lane 4 and 8). This could be due to incomplete knockdown efficiency of ATR, i.e., a small amount of ATR is sufficient for CHK1 phosphorylation but not for RPA2 phosphorylation under the tested conditions.

### HERC2 ubiquitinates and downregulates Ser33-phosphorylated RPA2

**Figure 4:**
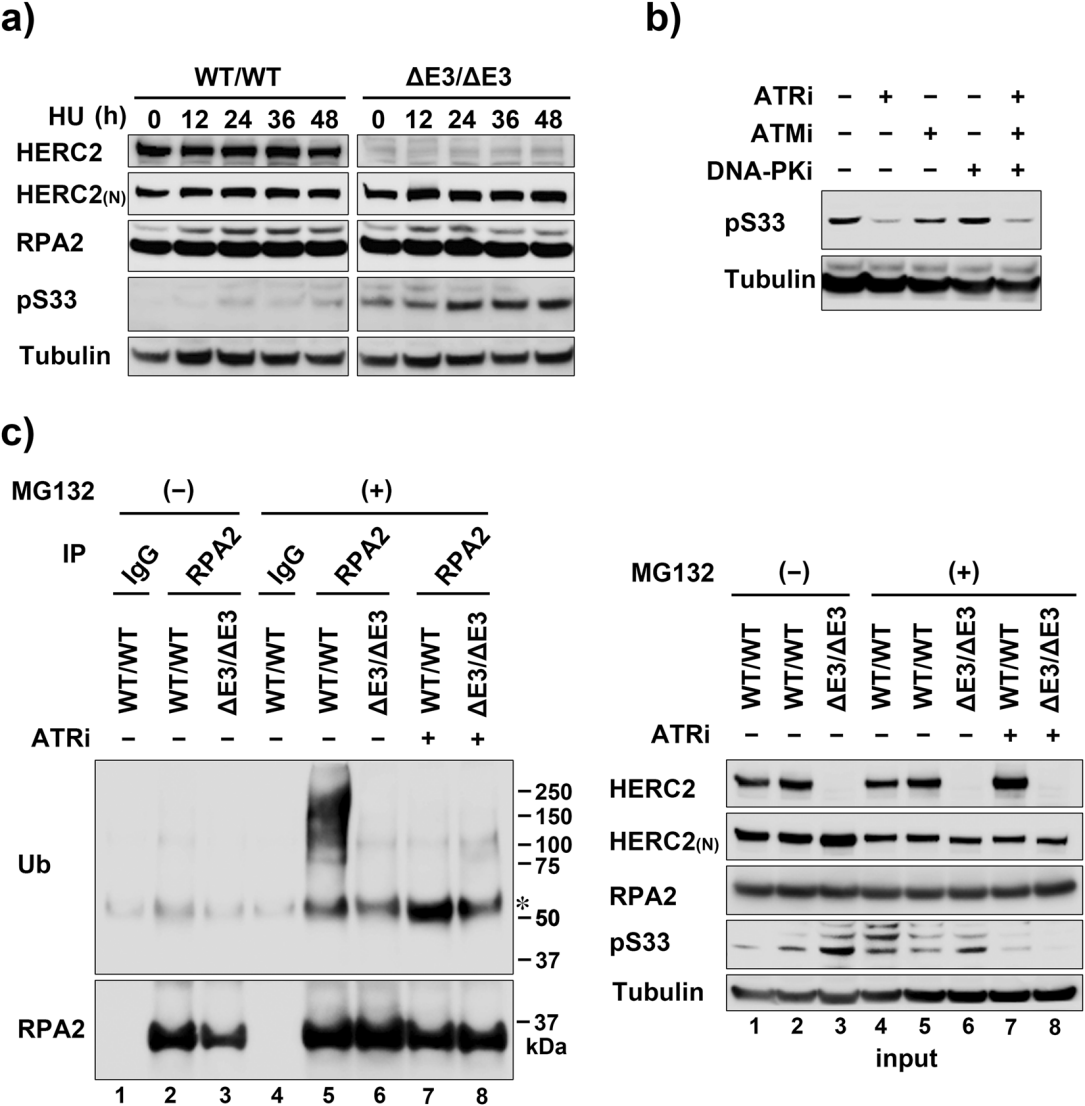
Effect of suppression of HERC2 E3 ligase activity on RPA2 Ser33 phosphorylation and ubiquitination. (**a**) Wild type and HERC2^ΔE3/ΔE3^ HCT116 cells were treated with 0.2 mM HU for the indicated time and subjected to immunoblotting with the indicated antibodies. HERC2 _(N)_: the antibody to an epitope 1781–1974 of HERC2. (**b**) HCT116-HERC2^ΔE3/ΔE3^ cells were incubated with or without 10 μM ATR inhibitor VE821, 10 μM ATM inhibitor Ku55933, or 10 μM DNA-PK inhibitor Nu7026 for 2 h as indicated and subjected to immunoblotting with the indicated antibodies. (**c**) Wild type and HERC2^ΔE3/ΔE3^ HCT116 cells were treated or not with MG132 and 5 μM ATR inhibitor for 12 h as indicated and subjected to immunoprecipitation in denature condition with control IgG or anti-RPA2 antibody followed by immunoblotting (left panels) or to direct immunoblotting (right panels).

To investigate the specific role of HERC2 E3 activity on phosphorylated RPA2, we next used HCT116 cells with CRISPR/Cas9-mediated homozygous deletion of C-terminal catalytic ubiquitin-binding site of HERC2 (HCT116-HERC2^ΔE3/ΔE3^). HERC2^ΔE3/ΔE3^ lacks amino acid residues 4758–4834 including the ubiquitin-binding site Cys4762 but expressed at the same level as wild type and is capable of interacting with helicases and RPA^20^. We first examined RPA2 Ser33 phosphorylation in the time course after adding 0.2 mM HU. Surprisingly, in contrast to HERC2 depletion, Ser33 phosphorylation was significantly enhanced by the HERC2 E3 inactivation, and even unstressed cells slightly expressed the phosphorylated RPA2 (Fig. 4a). Enhanced Ser33 phosphorylation was also observed early time (2–4 h) after MMC treatment (Fig. S4). ATR inhibitor, but neither ATM inhibitor nor DNA-PK inhibitor inhibited the RPA2 Ser33 phosphorylation in unstressed HCT116-HERC2^ΔE3/ΔE3^ cells (Fig. 4b), indicated increase of ATR-mediated phosphorylated RPA2 products in cells without HERC2 E3 activity. We then investigated effect of MG132 and ATR inhibitor on RPA2 Ser33 phosphorylation coupled with RPA2 ubiquitination status. Wild type and HERC2^ΔE3/ΔE3^ cells were treated with or without MG132 and ATR inhibitor, and subjected to either detection of RPA2 ubiquitination (Fig. 4c, left panels), or direct immunoblotting (input, right panels). Treatment with MG132 allowed ubiquitinated RPA2 product to be detected in wild-type cells that was inhibited in HERC2^ΔE3/ΔE3^ cells (left panel, lane 5 and 6). Increased level of Ser33-phosphorylated RPA2 was again detected in HERC2^ΔE3/ΔE3^ cells (right panel, lane 3). The increased level of Ser33-phosphorylated RPA2 was also detected in wild-type cells with MG132 treatment (right panel, lane 4 and 5), but it was not further increased in HERC2^ΔE3/ΔE3^ cells (lane 6), suggesting that the increased Ser33-phosphorylated RPA2 is due to deficiency of HERC2-mediated proteasomal degradation. Inhibition of the Ser33 phosphorylation by ATR inhibitor (right panel, lane 7 and 8) abolished the ubiquitination of RPA2 in wild-type cells (left panel, lane 7), further supporting the phosphorylation dependent substrate specificity. Note that the steady state level of RPA2 was unchanged in this condition, indicated that the ubiquitination and degradation of RPA2 occurs only a small fraction of RPA2 as is the case for RFWD3-induced ubiquitination^24^.

### RPA is epistatic to BLM and WRN in G4-suppressing function

**Figure 5:**
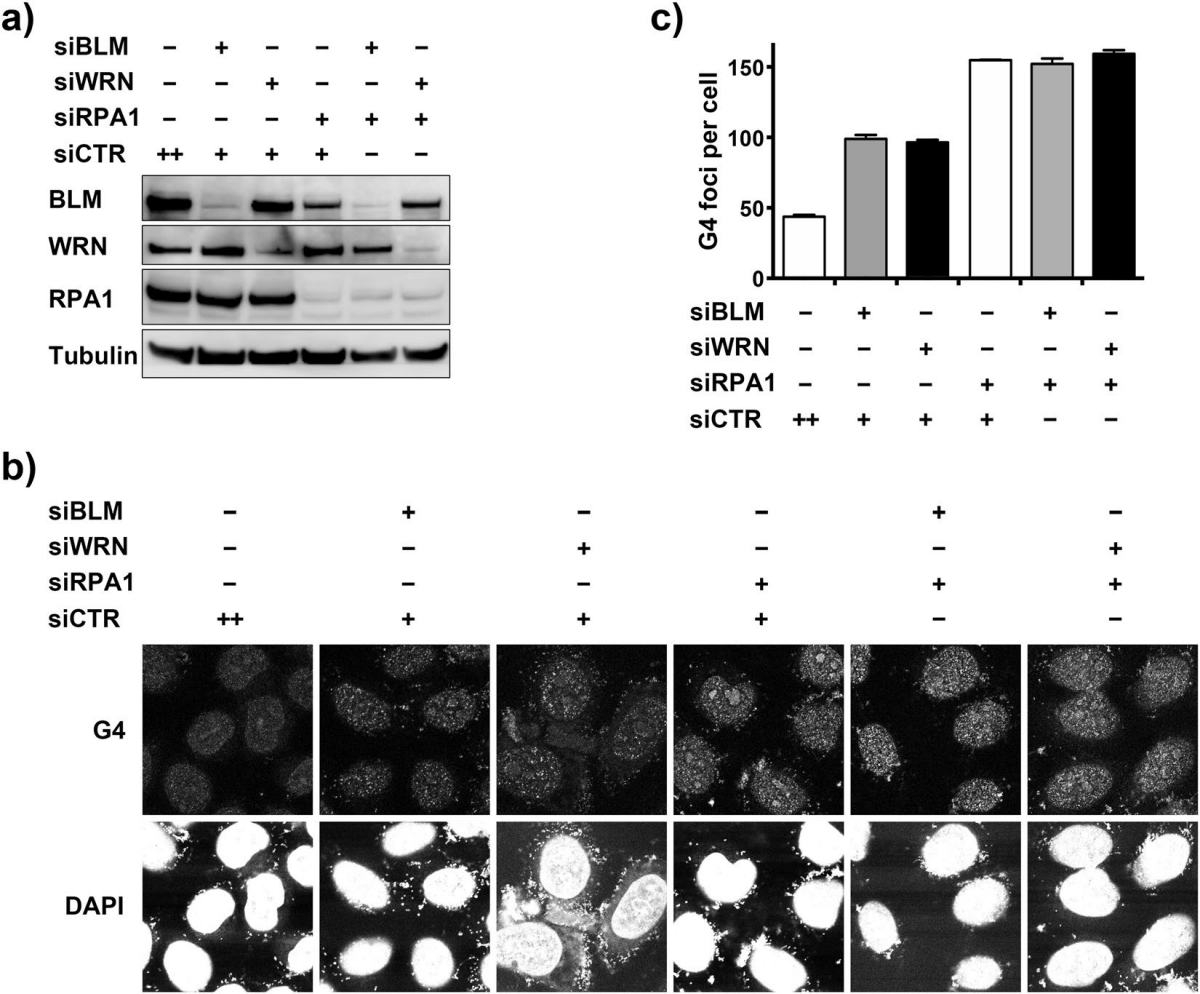
Epistatic relationship of BLM, WRN, and RPA1 on G4 formation. (**a**,**b**) HeLa cells were transfected with either control siRNA (CTR) or siRNAs specific to BLM, WRN or RPA1 as indicated and subjected to immunoblotting with the indicated antibodies (**a**) or immunostained for G4 (**b**). (**c**) Quantification of the mechanically counted G4 foci per cell is shown. Error bars, SEM of duplicate experiments, each based on more than 100 cells.

HERC2 suppresses G4 in a manner epistatic to the additive effects of BLM and WRN^20^. Because HERC2 was required for interaction of RPA with BLM and WRN complexes and regulates the turnover of RPA2 in the complexes via E3 activity, RPA may play a key role in the HERC2-mediated G4 suppression via BLM and WRN. *In vitro*, it has been reported that RPA alone or in collaboration with BLM and WRN, is capable of suppressing G4^26–29^. However, effect of RPA depletion on G4 accumulation in cells has not been investigated, especially in the context of epistatic relationship with BLM and WRN. To clarify this point, we first inhibited RPA1 and examined G4. Knockdown of either RPA1 or RPA2 inhibited RPA1 expression at similar extent (Fig. S5a) as previously reported^30^, indicating that each knockdown induces the same effect on RPA function. Depletion of RPA1 resulted in accumulation of G4 as expected (Fig. S5b–d). We next inhibited the expression of BLM or WRN with or without concomitant depletion of RPA1. The efficient depletion of each protein was confirmed by immunoblotting (Fig. 5a). Depletion of each single protein demonstrated that RPA has a dominant effect for G4 suppression when compared to BLM or WRN (Fig. 5b,c). Importantly, additional depletion of BLM or WRN in the RPA1-depleted cells did not further enhance the accumulation of G4 (Fig. 5b,c). The result suggests that RPA is epistatic to BLM and WRN in G4-suppressing function, reminiscent to the relationship of HERC2 with BLM and WRN^20^.

### HERC2 and its E3 activity are epistatic to RPA in G4-suppressing function

**Figure 6:**
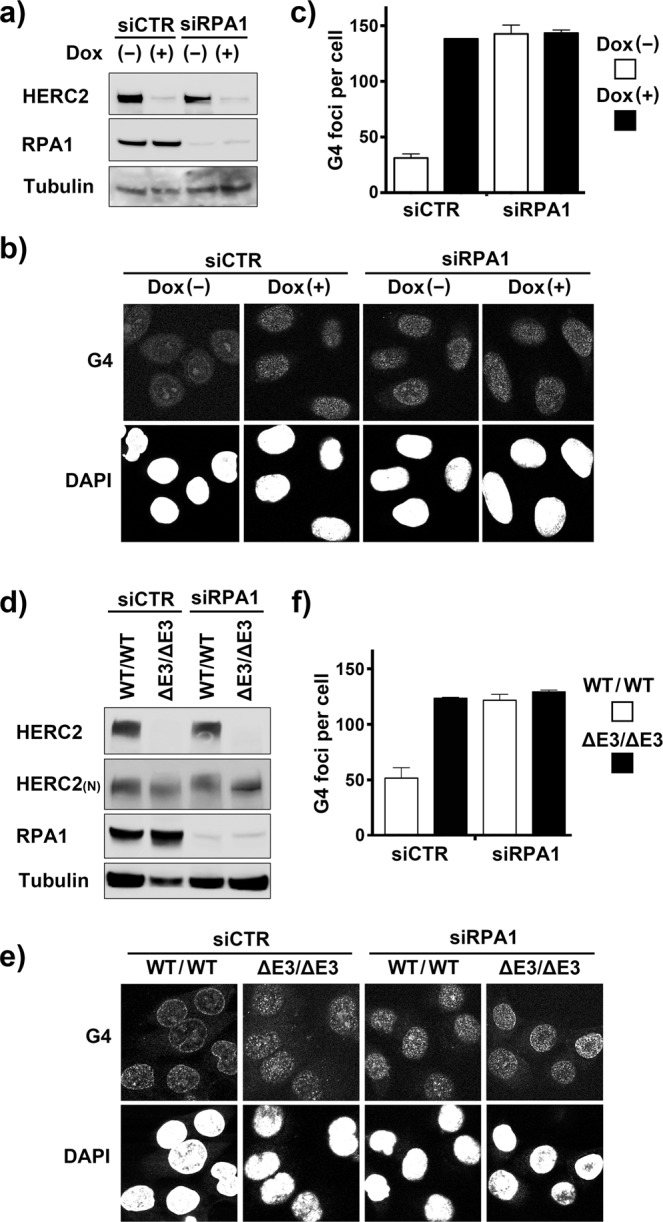
Epistatic relationship of RPA1 and HERC2 or its E3 activity on G4 formation. (**a**,**b**) HeLa-shHERC2 cells were transfected with the indicated siRNAs, induced or not with Dox and subjected to immunoblotting with the indicated antibodies (**a**) or immunostained for G4 (**b**). (**c**) Quantification of the mechanically counted G4 foci per cell is shown. Error bars, SEM of duplicate experiments, each based on more than 100 cells. (**d**,**e**) Wild-type or HERC2^ΔE3/ΔE3^ HCT116 cells were transfected with the indicated siRNAs and subjected to immunoblotting with the indicated antibodies (**d**) or immunostained for G4 (**e**). (**f**) Quantification of the G4 foci was performed as in (**c**).

The epistatic relationship of RPA with BLM and WRN in G4-suppressing function, together with similar relationship of HERC2 with BLM and WRN^20^ prompted us to test the relationship between HERC2 and RPA for G4 suppression. HeLa-shHERC2 cells were transfected with control or RPA1-specific siRNA and induced or not with Dox. The effective depletion of each protein was verified by immunoblotting (Fig. 6a). The depletion of HERC2 resulted in higher level of G4 accumulation (Fig. 6b,c) than that observed in BLM- or WRN-depleted cells, consistent with our previous observation that HERC2 is epistatic to the additive effects of BLM and WRN^20^. Importantly, this effect was approximately at the same level as that induced by RPA1 depletion, and simultaneous depletion of both proteins did not further elevated the level of G4 accumulation in each single depletion (Fig. 6b,c), indicating epistatic relationship between HERC2 and RPA on the G4 suppressing function.

To further investigate whether E3 activity of HERC2 is epistatic to RPA in G4-suppressing function, we next employed HCT116-HERC2^ΔE3/ΔE3^ cells. Wild type or HERC2^ΔE3/ΔE3^ cells were transfected with control or RPA1-specific siRNA. The depletion of RPA1 was verified by immunoblotting (Fig. 6d). The level of G4 accumulation by depletion of RPA1 in wild type HCT116 cells was approximately at the same level as that in HCT116-HERC2^ΔE3/ΔE3^ cells with control siRNA, and depletion of RPA1 did not further elevated the level of G4 accumulation in the HCT116-HERC2^ΔE3/ΔE3^ cells (Fig. 6e,f). Together, these results suggest epistatic relationship between E3 activity of HERC2 and RPA function that are also epistatic to the functions of BLM and WRN on G4 suppression.

## Discussion

Thus far, two E3 ligases, RFWD3 and PRP19, have been found to ubiquitinate RPA^24,25^. RFWD3 is a Fanconi anemia protein; a mutation in this protein in Fanconi anemia patients interferes with ubiquitination of RPA and RAD51^24,31–33^. PRP19 is a regulator of pre-mRNA splicing and functions in RNA maturation^34^. Both RFWD3 and PRP19 ubiquitinate hyperphosphorylated RPA2 in response to genotoxic stress and play critical roles in repair at stalled replication forks, homologous recombination, and interstrand crosslink repair (ICLR)^24,25,32,33,35^. However, ubiquitination mediated by PRP19 is not involved in protein degradation, but rather provides a scaffold for the recruitment ATR^25,35^. Ubiquitination mediated by RFWD3 does not cause RPA degradation after brief replication stress^24^, although it causes RPA2 degradation after prolonged exposure to MMC^33^. In the present study, we provide evidence that supports the hypothesis that HERC2 is the third E3 ligase for RPA2 and targets RPA2 for degradation.

Although HERC2, RFWD3, and PRP19 share some common functions involving RPA, they exhibit apparent differences in other functions, as summarized in Table 1. While RFWD3 constitutively binds RPA and maintains the interaction at stalled replication forks after stress^24,32,33,35–37^ and PRP19 only recognizes hyperphosphorylated RPA after DNA damage^25,35^, HERC2 constitutively interacts with RPA and dissociates from RPA after such severe damage^20^. RFWD3 and PRP19 interact with ssDNA-bound RPA, whereas HERC2 does not anchor to RPA-coated ssDNA^20^. RFWD3^24,36^ and PRP19^25^ are required for hyperphosphorylation, i.e., phosphorylation of RPA2 Ser33, Ser4/8, and Thr21. In contrast, HERC2 is required for Ser33 phosphorylation after low-level replication stress but not for Ser4/8 or Thr21 phosphorylation after more severe damage. Because Ser33 phosphorylation by ATR promotes phosphorylation of Thr21 and Ser4/8 by DNA-PK and ATM during DNA replication^9–12^, HERC2 is likely required for this early process of mild replication disturbance such as that induced by G4. HERC2 does not contribute to CHK1 phosphorylation; rather, it suppresses it through USP20 and Claspin degradation soon after stress^38,39^, while RFWD3 soon after stress^24,37^ and PRP19^25^ facilitates CHK1 phosphorylation. Importantly, E3 inactivation of HERC2, which preserves the interaction with RPA, increased Ser33 phosphorylation in the present study. Such characterizations for RFWD3 and PRP19 have not been performed previously. While RFWD3 and PRP19 ubiquitinate hyperphosphorylated RPA2 after genotoxic stress^24,25,32,33,35^, HERC2 ubiquitinates Ser33-phosphorylated RPA2 without exogenous stress. Most obviously, RFWD3-mediated ubiquitination of RPA2 after HU treatment is enhanced by an ATR inhibitor accompanied by increased Ser4/8 and Thr21 phosphorylation^24^, suggesting fork breakage–dependent, ATR-independent ubiquitination of hyperphosphorylated RPA2, whereas HERC2 mediated ubiquitination is inhibited by an ATR inhibitor. Furthermore, PRP19-mediated ubiquitination is non-degradative and rather provides a platform for protein recruitment^25,35^. Similarly, RFWD3-mediated ubiquitination is non-degradative and MG132 even suppresses this ubiquitination^24^, although it signals for degradation at a late stage in ICLR^33^. In our study it is likely that RFWD3 facilitates degradation of RPA2 in cells treated with 1 mM HU (Fig. 3g). The discrepancy between our result and the previous study using ssDNA damaging agents including HU^24^ is possibly caused by length of the stress. We treated the cells for 16 hours whereas cells were treated for one or two hours in the previous study^24^. In contrast, HERC2-mediated ubiquitination signals for degradation even in less-stressed cells, which can only be detected when cells are treated with MG132. Finally, while depletion of RFWD3 resulted in failure of HR, fork restart, and ICLR^24,31–33^, and depletion of PRP19 resulted in failure of HR and fork restart^25,35^, depletion of HERC2 or inhibition of its E3 activity enhances sister chromatid exchange (SCE)^20^, which is a consequence of HR, and increases G4^20^. Altogether, these data indicate separate roles for these three E3 ligases in RPA and DNA replication. One possible model is that HERC2 maintains DNA replication by protecting secondary structures of DNA including G4 arising from natural processes through RPA2 phosphorylation and subsequent removal of RPA while suppressing CHK1 activation, whereas RFWD3 and PRP19 handle higher order of DNA damage after stalled fork in collaboration with other PIKKs and CHK1 in addition to ATR. Furthermore, recent study suggests an essential role for RFWD3, which interacts with PCNA, on replication in unperturbed cells, suggesting more abundant functions of RFWD3 at replication fork^40^.

**Table 1:** Comparison of the main features of RFWD3, PRP19, and HERC2.

	RFWD3	PRP19	HERC2
RPA interaction timing	Constitutive At stalled fork	After genotoxic stress Hyperphosphorylated RPA2	Constitutive Dissociates after damage
RPA interaction situation	ssDNA-bound RPA	ssDNA-bound RPA	ssDNA-unbound RPA
Effect of knockdown on RPA2 phosphorylation	pS33↓, pS4/8↓, pT21↓(short) pS33↑, pS4/8↑ (long MMC)	pS33↓, pS4/8↓, pT21↓	pS33↓, pS4/8↓, pT21↓ (low stress) pS4/8→, pT21 → (high stress)
Effect of E3 domain deletion on RPA2 phosphorylation	NA	NA	pS33↑
Effect of knockdown on Chk1 phosphorylation	pS317-Chk1↓ (early after stress) pS317-Chk1→	pS345-Chk1↓	pS317-Chk1↑ (early after stress) pS317-Chk1→
RPA2 modification for ubiquitination	Hyperphosphorylated RPA2	Hyperphosphorylated RPA2	Ser33 phosphorylated RPA2
Trigger for ubiquitination	Genotoxic stress	Genotoxic stress	Without stress
Effect of ATR inhibition	Elevates RPA2 ubiquitination (after stress)	Dissociates from RPA	Inhibits RPA2 ubiquitination
Signal by ubiquitination	Non-degradation Degradation (long exposure to MMC)	Non-degradation	Degradation
Consequences of dysfunction	Failure in HR, fork restart, ICLR	Failure in HR, fork restart	Accumulation of G4, SCE

It is interesting that HERC2 displays paradoxical functions on RPA. Depletion of HERC2 inhibits RPA2 Ser33 phosphorylation and RPA interaction with BLM and WRN helicase complexes^20^. In contrast, HERC2^ΔE3/ΔE3^ cells exhibit increased Ser33 phosphorylation and accumulation of RPA in the helicase complex^20^. Such a paradoxical function may contribute to fine-tuning of the damage response. From this point of view, it is noteworthy that both phosphorylation and dephosphorylation of RPA2 are required for appropriate recruitment of RPA and repair factors in response to DNA damage^41,42^. In addition, ATR can induce rapid RPA exchange on ssDNA. Loss of ATR kinase activity dramatically reduces RPA exchange, resulting in accumulation of ATR with increased Ser4/8 and Thr21 phosphorylation of RPA2^43^. How HERC2 constitutively interacts with unphosphorylated RPA2, induces phosphorylation, and ubiquitinates and dissociates from RPA2 is currently unknown. HERC2 E3 ligase activity could be inhibited by auto-ubiquitination and oligomerization, the mechanism shared by large HECT E3 ligases, and remain poised for reactivation^21,22^ while carrying RPA. If this is the case, the trigger for reactivation remains a fascinating subject to elucidate.

The effects of Ser33-phosphorylated RPA2 and its degradation by HERC2 are currently not entirely clear. However, the possible epistatic relationship of HERC2 and its E3 activity with RPA in G4 suppression judged with siRNA knockdown experiments supports the hypothesis that the phosphorylation and degradation of RPA2 are critical functions of HERC2 in G4 suppression. We previously showed that HERC2 is also epistatic to additive effect of BLM and WRN for G4 suppression, although there is no epistatic relationship between BLM and WRN^20^. Together with the epistatic relationship between RPA and BLM and WRN, these results suggest that HERC2-BLM-RPA and HERC2-WRN-RPA share the role of G4 suppression. RPA has the ability to unfold G4 *in vitro* by itself or in combination with helicases^26–29^. HERC2 may support this collaborative function by supplying RPA and regulating its turnover.

In conclusion, we showed in the present study the essential role of HERC2 in regulating the cell response to replication stress and G4 suppression. Loss of this HERC2-dependent replication control could lead to genomic instability in HERC2-deficient cancers that may in turn provide opportunities for therapeutic strategies.

## Materials and Methods

### Cell lines and culture conditions

HeLa, HCT116, and HEK293T cells were obtained from ATCC with authentication and stored in liquid nitrogen or cultured according to the supplier’s instructions for less than 20 passages. All cells were routinely monitored for mycoplasma with a Mycoplasma Detection Set (TaKaRa). HeLa cells stably expressing HERC2-specific shRNA (5′-GAAGGTGGCTGTTCACTCA-3′) in a doxycycline (Dox)-inducible manner (HeLa-shHERC2) and HCT116 cells lacking the HERC2 catalytic ubiquitin-binding site (HCT116-HERC2^ΔE3/ΔE3^) due to CRISPR/Cas9-mediated insertion of the stop codon at E4758 were previously described^20^. HeLa-shHERC2 cells were treated with 1 µg/ml Dox for 48 h and subjected to individual experiments.

### Chemical agents

Chemical agents used in the present study were hydroxyurea (HU) (Sigma-Aldrich), mitomycin C (MMC) (Sigma-Aldrich), irinotecan hydrochloride (CPT) (Sigma-Aldrich), aphidicolin (APH) (Sigma-Aldrich), MG132 (Calbiochem), the ATR inhibitor VE821 (Toronto Research Chemicals), the ATM inhibitor Ku55933 (TOCRIS Bioscience), and the DNA-PK inhibitor Nu7026 (ChemScene).

### siRNAs, plasmids, and transfections

siRNA oligonucleotides targeting HERC2 (D-007180-02), ATR (S56826), RFWD3 (S30310), BLM (HSS101023), WRN (S14908), RPA1 (S12127), and non-targeting control siRNA (AM4635) were purchased from Ambion. RNA duplexes (final concentration 10 nM) were transfected into cells with Lipofectamine RNAiMAX (Invitrogen) 48 h before analysis. Fragments of human HERC2 with Myc-tag in a pcDNA3 plasmid were previously described^16^. Myc-HERC2-F6 and FLAG-HERC2-F7 were additionally subcloned into the pcDNA3 vector. A nuclear localization signal was generated in all HERC2 fragments. Six tandem HA-Ubiquitin repeats were constructed as previously described^44^. pcDNA3-St2-RPA1 and pcDNA3-St2-RPA2 were generated with RPA1 and RPA2 cDNA subcloned into pcDNA with tandem StrepII epitopes with a linker peptide that enables binding to Strep-Tactin with high affinity^45^. pcDNA3-HA-RPA2 was also subcloned with tandem HA epitopes. pEGFP-RPA1 and pcDNA3-FLAG-RPA3 were generous gifts from Dr. Minoru Takata of Kyoto University. Transfections of HEK293T cells were performed using the standard calcium phosphate precipitation method. Transfections of HeLa cells with simultaneous knockdown of HERC2 were performed with Lipofectamine 3000 (Thermo Fisher Scientific) according to the manufacturer’s instructions. Dox (1 μg/ml) was added immediately after transfection and cells were harvested 48 h later.

### Antibodies

Rabbit polyclonal antibody to C-terminal HERC2 (residues 4389–4834) was previously described^20^. The commercially available antibodies used in the present study were rabbit polyclonal antibodies against HERC2 (epitope 4784–4834, Bethyl Laboratories, A301-905A), RPA1 (Bethyl Laboratories, A300-241A), BLM (Bethyl Laboratories, A300-110A), WRN (Bethyl Laboratories, A300-239A), ATR (Bethyl Laboratories, A300-138A), RFWD3 (abcam, ab99306), Phospho-S345 Chk1 (Cell Signaling, 2341), Phospho-S317 Chk1 (R&D Systems, AF2054), RPA2 (Bethyl Laboratorries, A300-244A), Phospho-S33 RPA2 (Bethyl Laboratories, A300-246A), Phospho-S4/8 RPA2 (Bethyl Laboratories, A300-245A), Phospho-T21 RPA2 (abcam, ab61065), and mouse monoclonal antibodies against HERC2 (epitope 1781–1974, BD Biosciences, 17), StrepII (Sigma-Aldrich, SAB2702215), Chk1 (Santa Cruz Biotechnology, G4) α-tubulin (Neomarkers, DM1A), Myc (BabCo, 9E10), FLAG (Sigma-Aldrich, M2), HA (Boehringer, 12CA5), RPA2 (Calbiochem, RPA34-19), conjugated ubiquitin (NIPPON BIO-TEST Laboratories, FK2), and G4 (Absolute antibody, BG4).

### Immunoprecipitation and immunoblotting

Cell lysates were prepared with 0.5% NP-40 buffer (50 mM Tris-HCl [pH 7.5], 0.5% Nonidet P-40, 150 mM NaCl, 50 mM NaF, 1 mM dithiothreitol, 1 mM NaVO3, 1 mM PMSF, 2 µg/ml aprotinin, 2 µg/ml leupeptin, 10 µg/ml trypsin inhibitor, and 150 µg/ml benzamidine, supplemented with 125 U/ml Benzonase nuclease [Novagen] and 2 mM MgCl2) followed by immunoprecipitation and immunoblotting as described previously [36]. For direct immunoblotting, total cell lysate was prepared with RIPA buffer (50 mM Tris-HCl [pH 7.5], 150 mM NaCl, 0.1% SDS, 0.5% sodium deoxycholate, 1% Triton X 100, 1 mM dithiothreitol, 1 mM NaVO3, 1 mM PMSF, 2 µg/ml aprotinin, 2 µg/ml leupeptin, 10 µg/ml trypsin inhibitor, and 150 µg/ml benzamidine). For detection of endogenous RPA2 ubiquitination, immunoprecipitation was carried out in denaturing conditions as previously described^44^ and immunoblotting was performed with anti-conjugated ubiquitin antibody. To detect ubiquitination of exogenous RPA2, cells were transfected with the indicated plasmids including St2-RPA2 and HA-ubiquitin, treated with or without 5 µM MG132 for 12 h, and harvested 48 h after transfection. Cells were lysed under denaturing conditions with 1% SDS-containing buffer and ubiquitinated RPA2 products were subjected to pulldown with Strep-Tactin and washed with a buffer with a high salt concentration (2 M NaCl) as previously described^45^ followed by immunoblotting with anti-HA antibody.

### Immunofluorescence microscopy

Immunostaining of G4 by mixed methanol–acetic acid fixation followed by permeabilization with Triton X-100 and RNase A was performed as previously described^20^. G4 nuclear foci were mechanically counted using the Cellomics Image Analyzer (Thermo Fisher), with the SpotDetector® BioApplication (Version 4, Cellomics). The threshold for the area and intensity of the foci was set as following. Channel 1, ObjectArea: 8,000-550,000, Channel 2, SpotAreaCh2: 10,000, SpotAreaIntenCh2: 32767, SpotTotalIntenCh2: 550,000,000,000.

## Supplementary information

Supplementary Figure S1 to S5

## Data Availability

The datasets generated during and/or analysed during the current study are available from the corresponding author on reasonable request.
