# Supplementary material for: HERC2 regulates RPA2 by mediating ATR-induced Ser33 phosphorylation and ubiquitin-dependent degradation
Source: Sci Rep. 2019 Oct 3;9:14257. doi: 10.1038/s41598-019-50812-x (PMC6776656; doi:10.1038/s41598-019-50812-x)
Supplement: Supplementary file 1 — Supplementary Figure S1 to S5 [file 41598_2019_50812_MOESM1_ESM.pdf]

Supplementary Information

HERC2 regulates RPA2 by mediating ATR-induced Ser33 phosphorylation and ubiquitin-dependent degradation

Yongqiang Lai, Mingzhang Zhu, Wenwen Wu, Nana Rokutanda, Yukiko Togashi, Weixing Liang and Tomohiko Ohta

Supplemental information includes 4 figures.

SUPPLEMENTAL FIGURES

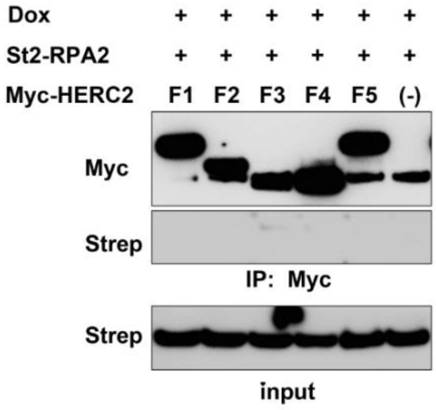

Figure S1. RPA2 does not interact with any fragments of HERC2 without MG132. HeLa-shHERC2 cells were co-transfected with the indicated HERC2 fragments and St2-RPA2, induced with Dox, and subjected to immunoprecipitation with anti-Myc antibody followed by immunoblotting with the indicated antibodies.

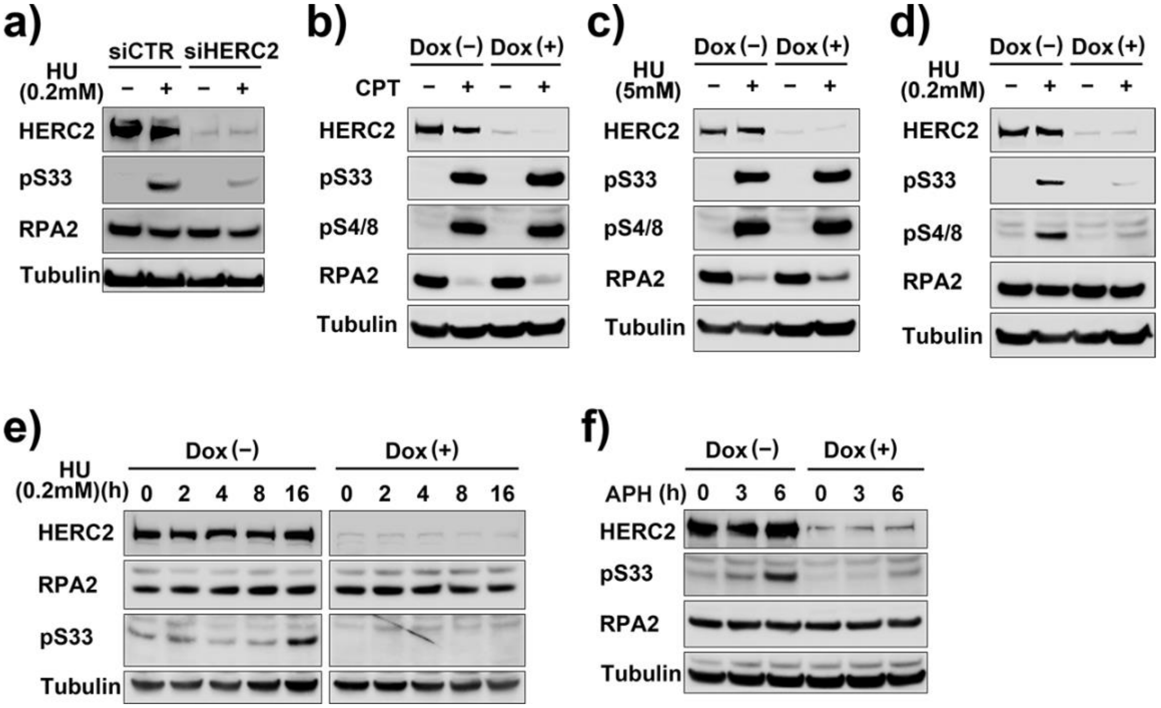

Figure S2. Depletion of HERC2 inhibited ATR-mediated phosphorylation of RPA2 induced by low-level replication stress.

(A) HeLa cells were transfected with the indicated siRNA, left untreated or treated with 0.2 mM HU, then subjected to immunoblotting with the indicated antibodies. (B to D) Effect of HERC2 depletion with a different shRNA. HeLa-shHERC2#2 cells harboring Dox-inducible shRNA with a sequence specific to HERC2 (5'-GGAAAGCACTGGATTCGTT-3') were induced or not with Dox, treated or not with the indicated genotoxic agents, and subjected to immunoblotting with the indicated antibodies. (E and F) HeLa-shHERC2 cells were left untreated or induced with Dox, then treated with 0.2 mM HU (E) or 5 µM APH (C) for the indicated time and subjected to immunoblotting with the indicated antibodies.

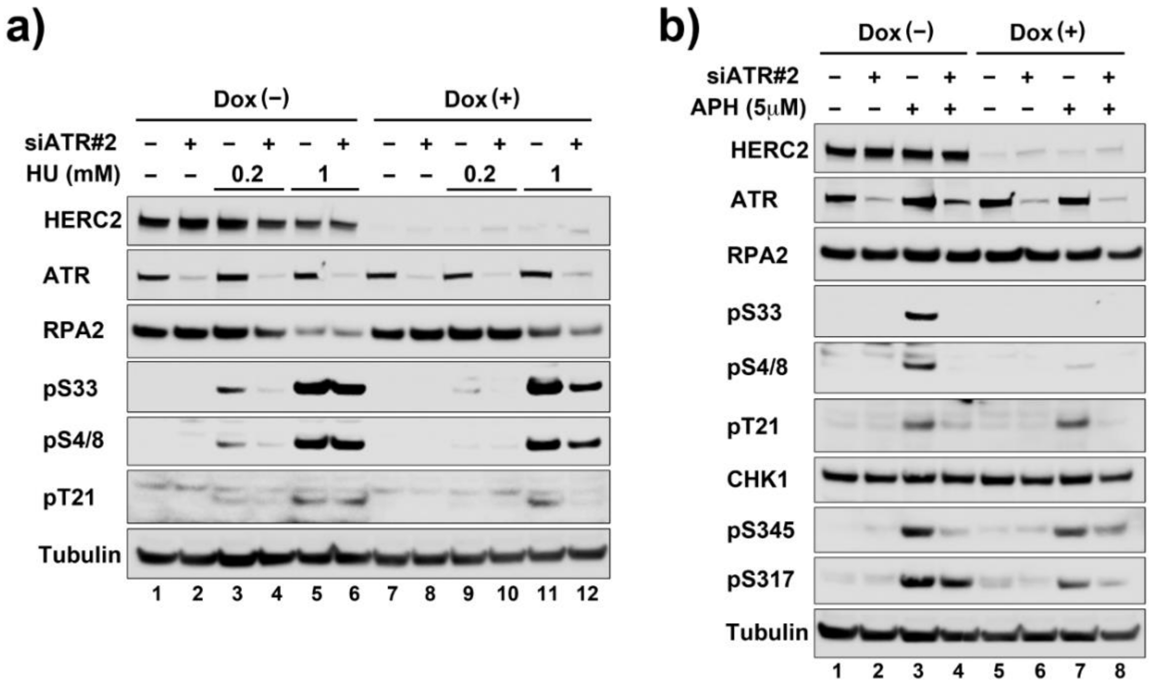

Figure S3. Depletion of HERC2 inhibit ATR-mediated phosphorylation of RPA2 induced by low-level replication stress.

Effect of HERC2 depletion on ATR-mediated phosphorylation of RPA2 shown in Fig 3E and F was reproduced with a different siRNA to ATR (siRNA#2, Ambion S57270). HeLa-shHERC2 cells were transfected with siRNA#2 specific to ATR, induced or not with Dox, treated or not with indicated concentration of HU (A) or APH (B), and subjected to immunoblotting with the indicated antibodies.

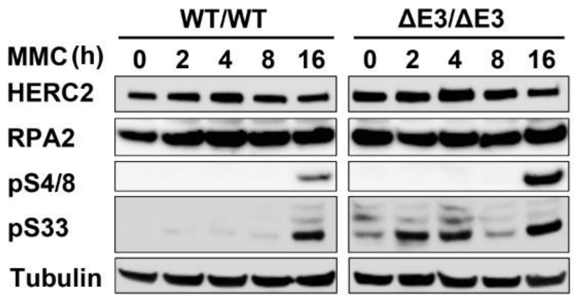

Figure S4. Effect of suppression of HERC2 E3 ligase activity on RPA2 Ser33 phosphorylation after MMC treatment.

(A) Wild-type and HERC2$^{\Delta E3/\Delta E3}$ HCT116 cells were treated with 0.5 μg/ml MMC for the indicated time and subjected to immunoblotting with the indicated antibodies.

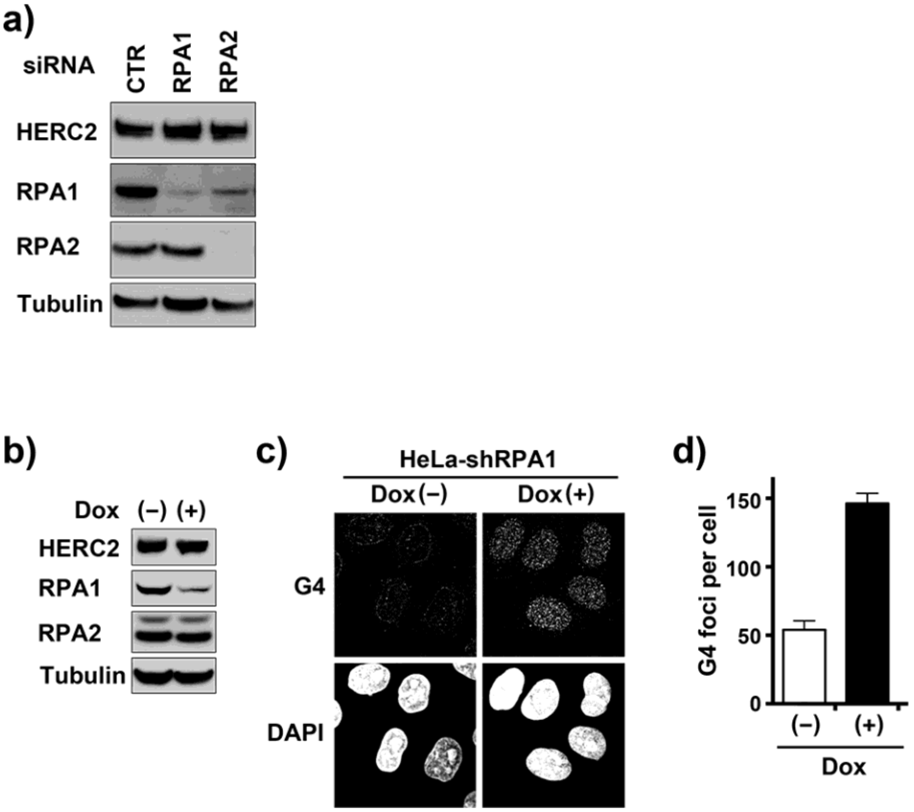

Figure S5. Effect of RPA1 depletion on G4 formation.

(A) HeLa cells were transfected with the indicated siRNAs and subjected to immunoblotting with the indicated antibodies. (B–D) HeLa-shRPA1 cells were induced with Dox or left untreated before being subjected to immunoblotting with the indicated antibodies (B) or immunostained for G4 (C). (D) Quantification of the mechanically counted G4 foci per cell. Error bars, SEM of duplicate experiments, each based on more than 100 cells.
